# Surgical and Functional Outcomes of Distal Femur Fractures Operated With Distal Femur Locking Compression Plate Versus Intramedullary Supracondylar Nail

**DOI:** 10.7759/cureus.83062

**Published:** 2025-04-27

**Authors:** Manish R Shah, Shyamal V Dave, Ankur Dahiya, Richenkumar R Shah, Shubham Nagar, Kunj R Patel

**Affiliations:** 1 Orthopaedics, Sumandeep Vidyapeeth Deemed to be University, Vadodara, IND; 2 Orthopaedics and Traumatology, Dhiraj Hospital, Vadodara, IND; 3 Orthopaedics and Traumatology, Sumandeep Vidyapeeth Deemed to be University, Vadodara, IND; 4 Orthopaedics, Smt. B. K. Shah Medical Institute & Research Centre, Vadodara, IND

**Keywords:** complex distal femur fracture, dflcp, distal femur fracture, imscn, inter-condylar femur fracture, supra-condylar femur nailing

## Abstract

Introduction

Distal femur fractures are common but challenging as they are usually displaced and comminuted. They are prone to functional impairment of the knee joint because of injury to the quadriceps system. Such fracture often occurs in elderly patients with osteoporosis. Various treatment methods, such as closed traction, application of a cast brace following preliminary traction, and open reduction with internal fixation using various implants, are suggested. We evaluated the surgical and functional results of distal femur fractures treated with anatomical distal femur locking compression plate (DFLCP) versus intramedullary supracondylar nail (IMSCN).

Materials and methods

The study was conducted from April 2023 to August 2024. We included 20 patients with distal femur fractures, who were planned to be operated on with DFLCP or IMSCN. They were followed up for at least six months. Surgical and functional outcomes were analyzed as per the Schatzker and Lambert criteria.

Results

DFLCP was preferred for inter-condylar (intra-articular) distal femur fractures. The average blood loss for the DFLCP group was 425 ml, and for the IMSCN group, it was 242.85 ml. The functional outcomes were comparable for both groups. The average union time for the DFLCP group was 8.15 months, and for the IMSCN group, it was 7.15 months. We analyzed the results by the Schatzker and Lambert criteria. We achieved 46% and 42.85% excellent results in plate v/s nail groups, respectively.

Conclusions

DFCLP is a better implant for inter-condylar, i.e., intra-articular and highly comminuted distal femur fractures. It gives anatomic and stable fixation. The average intraoperative blood loss is higher with DFLCP. The average union time is less with the IMSCN. Closed reduction, preserving fracture hematoma, and less soft tissue compromise result in early fracture union in IMSCN. Soft tissue compromise in open grade 3B fractures affects the functional outcomes. The final results do not change with the use of DFCLP or IMSCN.

## Introduction

Fractures in the distal femur have posed considerable therapeutic challenges. They are readily deformed because of muscle forces acting on the distal fragment and are frequently comminuted. Because of injury to the quadriceps system, they are prone to functional impairment of the knee and ankle joints. Such fractures are seen in young adults due to high-velocity accidents and low-velocity domestic falls in the elderly [1].

Distal femoral fractures account for 6% of femur fractures and approximately 10% of proximal femoral fractures [2]. The most common high-energy mechanism of injury is a road traffic accident (RTA) (53%), and the most common low-energy mechanism is a simple fall (33%). The overall incidence of distal femoral fractures is approximately 8.7/100,000/year [3]. These potentially serious injuries result in various degrees of permanent disability and continue to pose a therapeutic challenge to orthopedic surgeons even today in achieving a successful outcome. Different modalities of treatment, like closed traction, simple immobilization, and fixation with various implants, are advocated by different people.

Closed treatment results in long-term hospitalization and morbidity associated with prolonged recumbency. It is associated with shortening, malunion, and knee stiffness. Such issues led to the development of open reduction and internal fixation with implants. Osteoporosis adds to issues like poor fixation and loss of reduction for the fractures involving an articular surface. Achieving full knee joint movement is an issue as the joint is near the fracture [4]. Proper articular reduction, restoration of limb alignment, and early mobilization are effective ways of treating distal femoral fractures.

The principle of management includes axial alignment, anatomic restoration, and rotational stability of a fracture. Fixed-angle devices like angle blade plates and dynamic condylar screw need reasonable bone stock. The condylar buttress plate, used for comminuted fractures, faced a problem of varus collapse due to the toggle of the plate-screw interface. A locking plate system was devised to solve the issue. Such a system preserves the periosteal blood supply, too. An anatomical distal femur locking compression plate (DFLCP) properly fits the shape of the distal femur and gives a stronger lateral construct. The locking mechanism avoids a toggle at the plate-screw interface, so it is useful for osteoporotic bones [5-8]. Plates with combined holes have the advantage of fixing simple screws and locking screws. The anatomical shape of the plate does not need contouring, so it increases the purchase of the implant [9].

Extra-articular and partial articular fractures can be fixed by an intramedullary nail. An intramedullary device aligns the femoral shaft with condyles, reducing the tendency to place varus movement at the fracture site. The reduced bending movement of an intramedullary device has substantially reduced the fixation failure in osteoporotic bone. The anatomical alignment, stable internal fixation, rapid mobilization, and early functional rehabilitation of the knee are effective ways of managing distal femoral fractures, which can be achieved by intramedullary interlocking nails [10,11]. The use of such a nail can allow fracture fixation with limited soft tissue damage and preservation of fracture hematoma [12,13]. Also, the nail is a weight-sharing device, and the plate is a weight-bearing device. So, early weight bearing and mobilization are significant advantages of nails. Nail entry point by patellar tendon splitting approach can lead to damage to the knee joint and patella articular surface [14].

Though both implants have been used widely for such types of fractures, the superiority of one implant over the other is still unclear. In this study, we aimed to study the surgical and functional outcomes of distal femur fractures treated with DFLCP versus intramedullary supracondylar nail (IMSCN) and their complications. It is expected that the result of this study will help in choosing the proper implant for distal femur fractures.

## Materials and methods

After getting approval from the Ethical Committee of Sumandeep Vidyapeeth (Approval number: SVIEC/ON/MEDI/BNPG22/APRIL 23/281), we carried out a prospective observational study on patients admitted to our tertiary care medical college after obtaining written and informed consent. We aimed to study clinical and functional outcomes by two different modalities of treatment by objectively assessing intra-operative blood loss, hospital stay, time for union, and complications.

The study was conducted from April 2023 to August 2024 on 20 patients. All the patients who were operated on for distal femur fracture by open reduction internal fixation (ORIF) with DFLCP and closed reduction internal fixation with IMSCN, with an age of more than 18 years and with a minimum follow-up of six months, were included in the study. We included closed and open fractures (primarily managed with an external fixator) too. Patients with age less than 18 years, patients treated with other modalities of treatment, polytrauma patients with bilateral lower limb injuries or another injury in the same limb, fractures associated with hip or knee dislocations, patients with neurovascular complications, medically unfit patients, and those who refused to participate in the study were excluded.

All surgeries were done by three different senior professors (head of units) with more than 10 years of experience. All surgeries were done in three units of a tertiary care medical college set up. X-rays of the affected femur, AP and lateral views, were taken on admission. A computed tomography (CT) scan for pre-operative planning was done in a patient with intra-articular comminution. After shifting the patient to the ward, primary management till surgery was done with ankle traction of the affected limb and analgesics. All cases were subjected to routine pre-anesthetic check-ups, primary blood investigations, medical fitness (if required), and any additional investigations if necessary. Fractures were classified according to the AO (Arbeitsgemeinschaft für Osteosynthesefragen) classification for distal femur fractures. Patients with hemoglobin<10gm/dl were planned for intra and postoperative blood transfusions, particularly in the case of open reduction.

Fractures not having intra-articular extension (supracondylar fractures-type A fractures as per the AO classification) were preferred for IMSCNs. Those having intra-articular extension, comminuted fracture, and inter-condylar comminuted fractures (mainly AO classification type C) were planned for DFLCP.

Surgical technique

Surgical Technique for DFLCP

**Figure 1:**
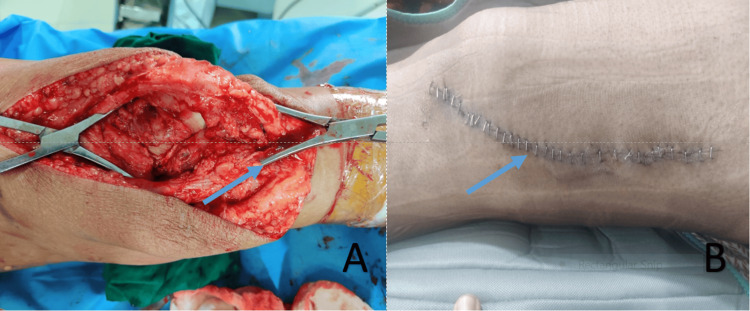
Swashbuckler approach (A) Intra-operative picture with an arrow pointing to intra-articular reduction. (B) Post-operative image with an arrow showing the line of the incision

After all aseptic and antiseptic precautions, all patients were operated on with the Swashbuckler approach to the distal femur (Figure 1). It allows surgical exposure of the entire articular surface of the distal femur. The patient was kept in the supine position with the knee flexed over a rolled or triangle pillow.

Surgical Technique for IMSCN

**Figure 2:**
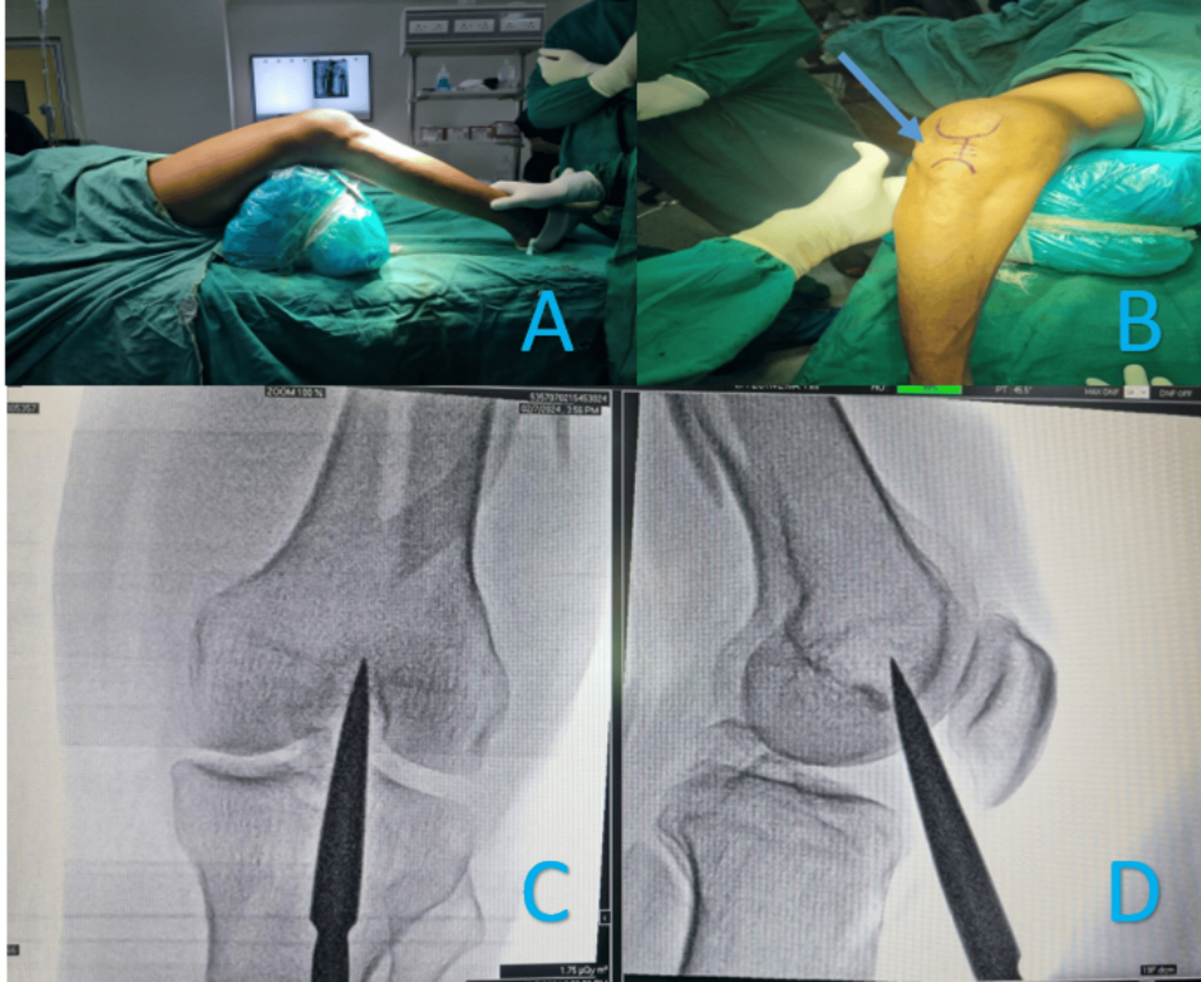
Technique for IMSCN (A) Position of the patient. (B) Incision shown by an arrow. (C) Intra-operative AP image. (D) Intra-operative lateral image IMSCN: Intramedullary supracondylar nail

Surgery was done on a radiolucent table in the supine position. The knee was in approximately 30° flexion with a triangular or rolled pillow underneath. A vertical incision of 3-5 cm was made in the midline from the lower pole of the patella. After the splitting of the patellar tendon, a sleeve was inserted. The entry was made in line with the medullary canal, just anterior to the femoral origin of the posterior cruciate ligament. The rest of the procedure was done as per the standard nailing protocol (Figure 2).

Intravenous antibiotics were given for 24 to 72 hours, and painkillers were continued till the patient was in the hospital. Quadriceps muscle strengthening exercise was taught. Once the wound condition was good, the patient was discharged (usually on the fifth post-operative day).

Suture removal was done between 12 and 15 days after surgery. Patients were followed up at one month, three months, and six months. X-rays were done until solid, continuous callus formation was observed. Patients were mobilized to non-weight-bearing walking with a walker immediately after surgery. Full weight bearing was started once radiological union was established during the subsequent follow-up visits. The radiological union was assessed by the presence of bridging callus in three out of four cortices in anteroposterior and lateral views. During each follow-up, the patient was assessed as per the Schatzker and Lambert criteria [15].

All data was entered in Microsoft Excel (Microsoft Corporation, Redmond, United States) and used for further analysis using IBM SPSS Statistics for Windows, Version 23 (Released 2015; IBM Corp., Armonk, New York, United States). We have presented data in numbers and percentages. For qualitative data generated during the study, we used the chi-square test to find the association between the variables. P-value <0.05 was considered a statistically significant value.

## Results

Patient demographics

Maximum (50%) patients were in the age group of 31-50; 35% of patients were younger age group (18-30 years). The remaining 15% were in the age group of 51-70 years. None of the patients was older than 70 years (Table 1). The mean age was 40.65 years.

**Table 1:** Age distribution DFLCP: Distal Femur Locking Compression Plate; IMSCN: Intramedullary Supra Condylar Nail

Age group (years)	DFLCP	IMSCN	Total (%)
18-30	4	3	7 (35%)
31-50	7	3	10 (50%)
51-70	2	1	3 (15%)
>70	0	0	0
Total	13	7	20
P-value: 0.8592			

In our study, most patients (75%) were male, as shown in Table 2.

**Table 2:** Gender distribution DFLCP: Distal Femur Locking Compression Plate; IMSCN: Intra Medullary Supra Condylar Nail

Gender	DFLCP	IMSCN	Total (%)
Male	9	6	15 (75%)
Female	4	1	5 (25%)
Total	13	7	20
P-value: 0.4197			

We found that most patients (65%) (13 out of 20) were operated on with DFLCP, and 35% (7 out of 20) were with IMSCN (p value=0.1797). The commonest plate (9/13) (69.23%) in the DFLCP group was 6, 7, and 9 holed (3 of each, respectively). A nail diameter of 11mm (5/7) (71.42%) was most commonly used, and the most common length was 250mm. RTAs were the most common cause of fracture (75%). Domestic fall was the cause in the remaining 25% of the patients. Distal femur fracture generally occurs due to high-velocity trauma. We found that the majority of patients had associated comorbidity of hypertension (HTN) (4/20). Only one patient had a combination of HTN and diabetes mellitus (DM), and one patient had rheumatoid arthritis.

Pre-operative analysis

In our study, only two patients had open (grade 3B) distal femur fractures remaining (18/20) were closed injuries (P-value=0.00035). We classified according to AO classification and found that patients of supracondylar (A1, A2, A3) (55%) and intercondylar (C1, C2, C3) (45%) fractures were almost equal as shown in Table 3. The maximum number of patients with inter-condylar fractures (8/9) (89%) were operated on with DFLCP.

**Table 3:** Fracture classification as per AO (Arbeitsgemeinschaft für Osteosynthesefragen) DFLCP: Distal Femur Locking Compression Plate; IMSCN: Intramedullary Supra Condylar Nail

Type	DFCLP	IMSCN	Total (%)
A1	2	3	5 (25%)
A2	2	3	5 (25%)
A3	1	0	1 (5%)
B1	0	0	0
B2	0	0	0
B3	0	0	0
C1	3	1	4 (20%)
C2	3	0	3 (15%)
C3	2	0	2 (10%)

Intra-operative analysis

In our study, the average blood loss was 242.85 ml for the IMSC nail group and 425 ml for the DFLCP group. Six patients had >400 ml of blood loss, and all were operated on with DFLCP. 7/20 (35%) had 200-300 ml blood loss. Only one patient had <200 ml blood loss and was operated on with an IMSC nail, as shown in Table 4.

**Table 4:** Intra-operative blood loss DFLCP: Distal Femur Locking Compression Plate; IMSCN: Intramedullary Supra Condylar Nail

Blood loss (ml)	DFLCP	IMSC	Total (%)
<200	0	1	1 (5%)
200-300	2	5	7 (35%)
300-400	5	1	6 (30%)
>400	6	0	6 (30%)
Total	13	7	20
P-value: 0.6547			

In our study, we found that four patients operated on for DFLCP needed intraoperative blood transfusion (BT), whereas only one patient of IMSCN needed intraoperative BT.

Post-operative analysis

In our study, radiological union time was almost similar in most patients in both groups, as shown in Table 5. The mean union time was 7.8 months. The mean union time was 8.15 and 7.15 months respectively for DFLCP and IMSCN groups, but it is not statistically significant (P-value: 0.8592).

**Table 5:** Radiological union time DFLCP: Distal Femur Locking Compression Plate; IMSCN: Intramedullary Supra Condylar Nail

Union time (In months)	DFLCP	IMSC	Total (%)
6 months	4	3	7 (35%)
6-9 months	7	3	10 (50%)
>9 months	2	1	3 (15%)
Total	13	7	20
P value: 0.8592			

**Figure 3:**
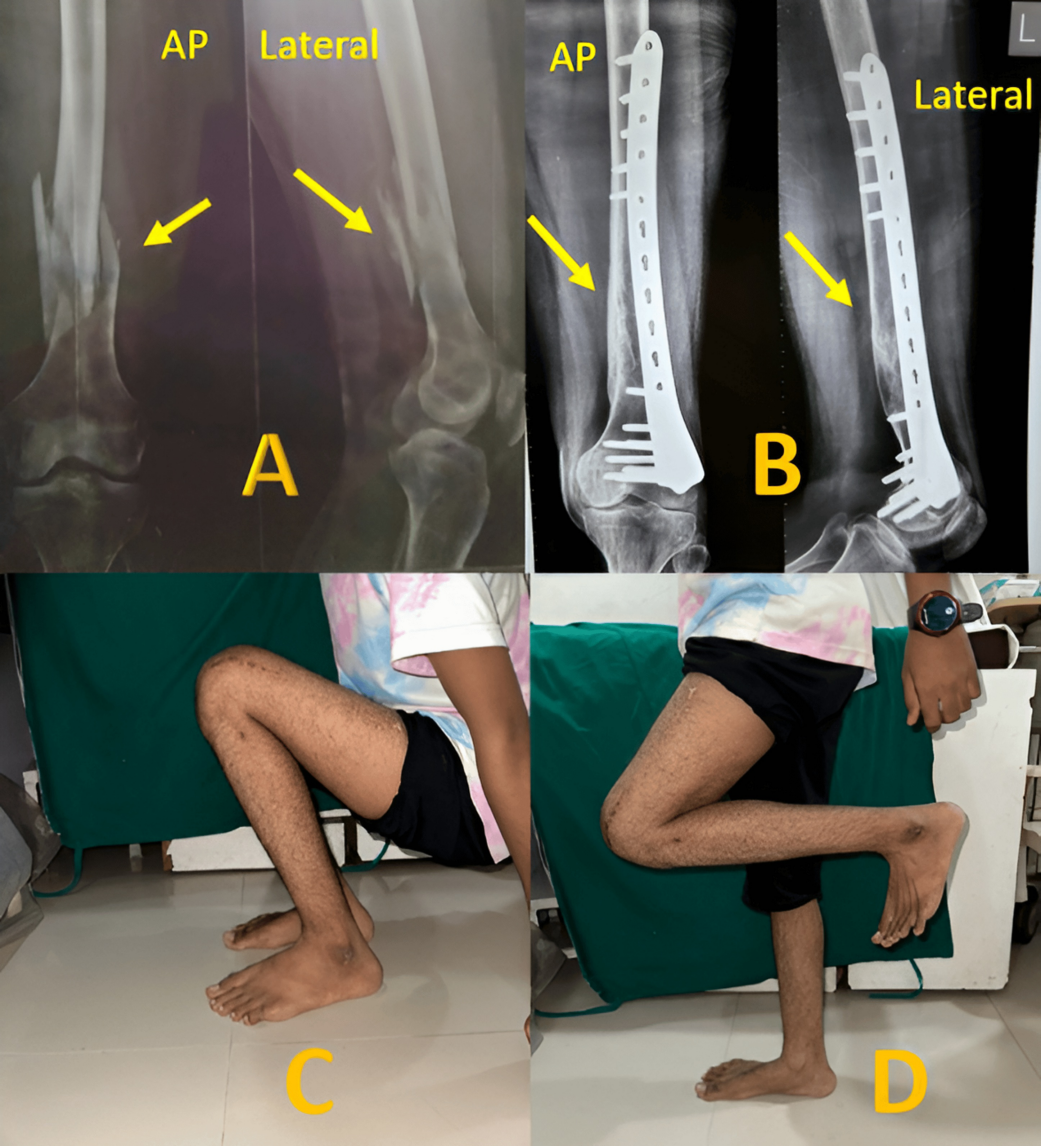
Excellent results obtained with DFLCP (A) AP and lateral X-rays after trauma (arrow showing comminuted fracture), (B) AP and lateral X-rays on the final follow-up (an arrow showing union), (C) Squatting at the final follow-up, and (D) Knee flexion at the final follow-up DFLCP: Distal Femur Locking Compression Plate

**Figure 4:**
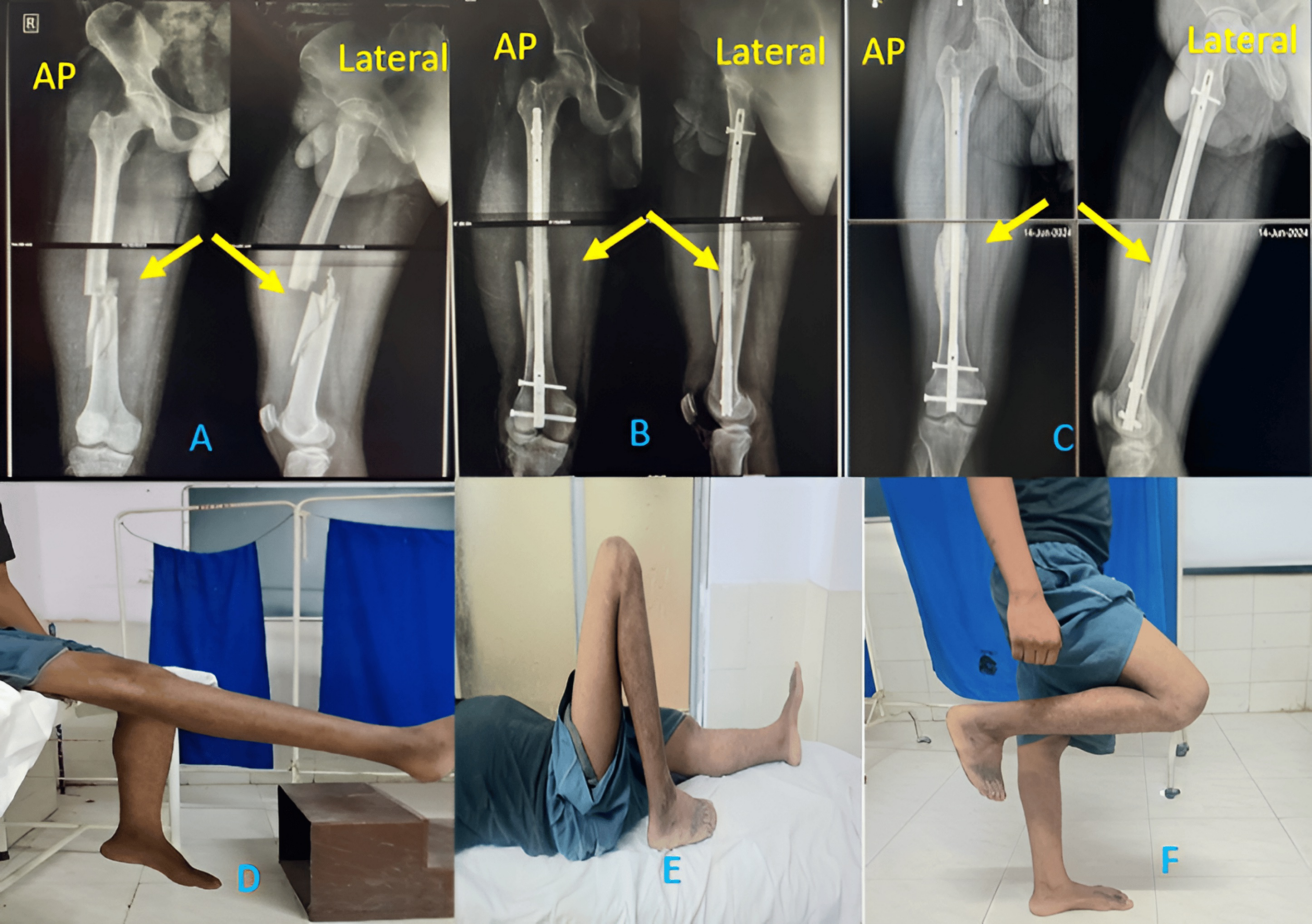
Excellent results obtained with IMSCN (A) AP and lateral X-rays after trauma (arrow showing fracture), (B) AP and lateral X-rays after the surgery (arrow showing the fracture), (C) AP and lateral X-rays at the final follow-up (arrow showing union at the fracture site), (D) Knee extension at the final follow-up, (E) Knee flexion at the final follow-up, and (F) Knee flexion in standing position IMSCN: Intramedullary Supracondylar Nail

In our study, most patients had excellent results (45%) (Figure 3), and only one had a poor result, as shown in Table 6. Almost half of the patients (6/13) operated with DFLCP had excellent results, out of which 3 had an inter-condylar distal femur fracture. Similarly, for IMSCN, 3/7 patients had excellent results (Figure 4), and all had supra-condylar distal femur fracture.

**Table 6:** Interpretation of Schatzker and Lambert criteria DFLCP: Distal Femur Locking Compression Plate; IMSCN: Intramedullary Supra Condylar Nail

Interpretation	DFLCP	IMSC	Total (%)
Excellent	6	3	9 (45%)
Good	3	3	6 (30%)
Fair	3	1	4 (20%)
Poor	1	0	1 (5%)
Total	13	7	20
P-value: 0.7247			

**Figure 5:**
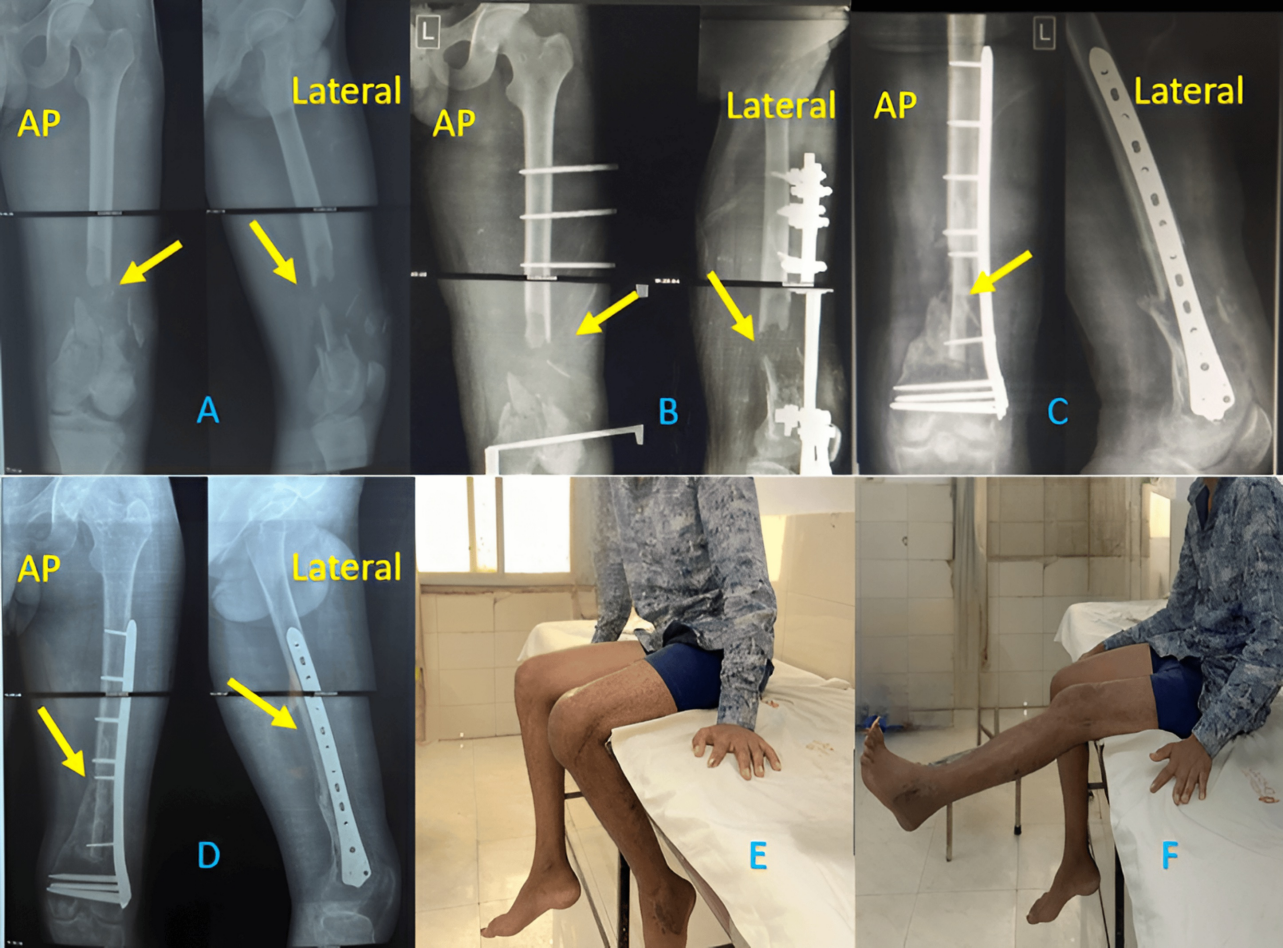
Results of DFLCP in open grade 3B fracture (A) AP and lateral views after the injury (arrow showing fracture), (B) AP and lateral X-rays after primary stabilization with an external fixator (arrow showing the fracture), (C) Post-operative X-rays after fixation with DFLCP and fibula strut graft (arrow showing the graft), (D) X-rays at the final follow-up (arrow showing well-united fracture), (E) Knee flexion at the final follow-up, and (F) Knee extension at the final follow-up DFLCP: Distal Femur Locking Compression Plate

One patient with open grade 3B (AO C3) distal femur fracture with bone defect was operated on with ORIF with DFCLP and fibula strut graft. He was primarily managed with an external fixator. This shows that DFLCP is a better implant choice for highly comminuted fractures with bone defects (Figure 5) [11].

**Figure 6:**
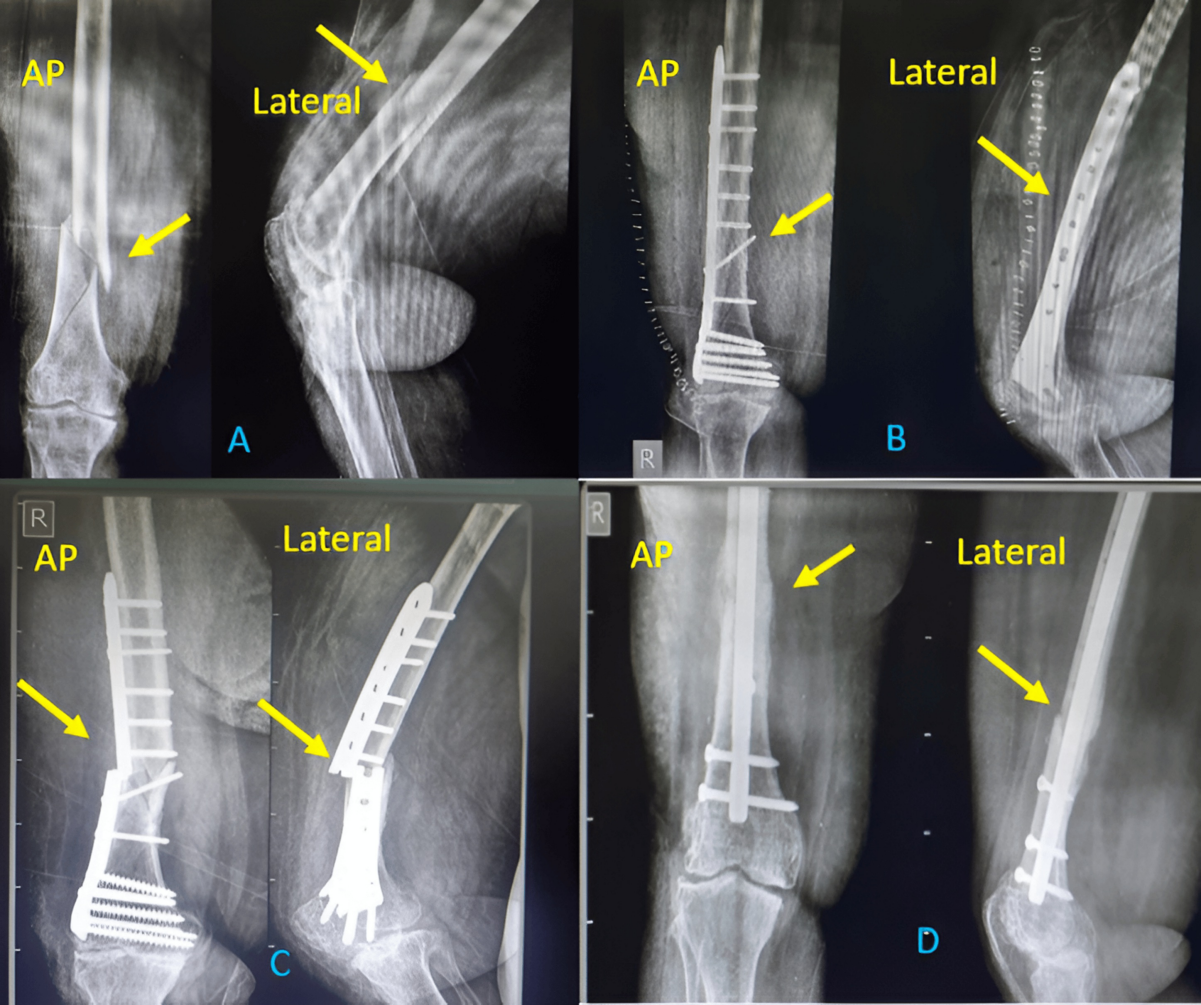
Poor results obtained with DFLCP (A) AP and lateral X-rays after trauma (arrow showing fracture), (B) AP and lateral X-rays after surgery (arrow showing fracture), (C) AP and lateral X-rays on follow-up (arrow showing broken plate), and (D) AP and lateral X-rays after revision surgery (arrow showing united fracture) DFLCP: Distal Femur Locking Compression Plate

We had one patient with a poor outcome in our study who was operated with DFCLP. She had uncontrolled DM and severe osteoarthritis with flexion deformity of the knee joint and was morbidly obese. The patient had delayed union and implant breakage. She was operated on with implant removal and fixation with the Sirus femur interlock nail. She was mobilized to toe touch weight bearing immediately after the second surgery and full weight bearing after four months of the second surgery (Figure 6).

## Discussion

Starting from the conservative treatment, various surgical options are available at present for the treatment of distal femur fractures. Supracondylar fractures of the femur are notoriously difficult to treat because they are highly unstable and comminuted. Because of strong muscular attachment, it is difficult to maintain it in proper alignment without fixation, and also because of their proximity to the knee, regaining full knee motion is usually more difficult. Nail is preferred for extra-articular fractures, i.e., supracondylar distal femur fractures [16-19]. The plate is the preferred implant for intra-articular, i.e., intercondylar fractures [20,21].

Locking plate fixation relies on principles of open reduction, absolute stability, and inter-fragmentary compression for bone healing. Osteosynthesis with DFLCP has the benefit of achieving near-anatomical fixation because of the direct reduction maneuver, which is necessary for intra-articular fractures [22] and comminuted fractures [23,24]. It is useful in the fixation of fragility fractures in osteoporotic bones [25]. Intramedullary nails are less invasive, achieve reduction via an indirect approach, and offer relative stability. IMSCN has the edge of being biological, not disturbing fracture hematoma, less soft tissue handling, and no loss of blood supply to the bone as the periosteum is left intact [13,26]. Its disadvantages are a lack of alignment control, posterior angulation, damage to the knee joint cartilage, risk of early osteoarthritis of the knee, anterior knee pain sometimes leading to implant removal, and the intra-articular effect of reaming debris. Also, it is difficult to achieve reduction with nails in intra-articular fractures [21]. With intramedullary nails being a sharing device, we can start immediate weight bearing depending on the fracture reduction and type of fracture.

Senthilnathan et al. [27] studied 30 patients with a mean age of 45.75. It was comparable to our study, with a mean age of 40.65. Yadav et al. [26] showed a male preponderance with a male-female ratio of 3:1 in plating and 4:1 in the nailing group. Said et al. [28] in their comparative study showed that the surgical technique affects clinical outcomes over the choice of implant. In their prospective study of 32 patients, the average intraoperative blood loss was 247 ml for nails and 454 ml for plating cases and was statistically significant. The average union time was three months for the nail group and 4.5 months for the plating group. There was no statistical difference in the knee movement between the two groups (P-value=0.346). The lower extremity functional score was used, and the nailing group had higher values. A study by Khan et al. supports the relation of post-operative rehabilitation and knee movement with the soft tissue injury [29].

Senthilnathan et al. [27] in their comparative study stated that nailing proved to have a good functional outcome in terms of early weight-bearing, knee flexion, and less union time. Both nailing and plating have excellent results with proper preoperative planning. In their study of 30 patients, the union time was 3.5 months for nails and four months for plating. Neer’s scoring system was used, which was higher for nails with 46% excellent results and 26% excellent, and 53% good results for plating. Early weight bearing was started in nail patients, and a common complication of anterior knee pain was seen in almost 50% of patients.

Krishna et al. [20] did a comparative study of 40 patients. A group of patients with distal femoral locking plates has a union time of 18.4 weeks (4.5 months), while distal femoral nail has a union time of 15.2 weeks (3.75 months). The p-value was 0.007, which is significant. This value shows that the distal femoral nail has a shorter union time. The average knee flexion was found to be 104 degrees for the nail and 110 degrees for the plating group and was not significant. They concluded that plate can be adapted to all fractures, while retrograde nailing is better adapted to extra-articular fractures.

Our results were comparable to the studies concerning the radiological union time [17,27]. IMSCN patients had shorter union time as compared to DFCLP patients. Our study also confirmed that the difference was statistically insignificant. Early weight bearing, closed reduction, preservation of the fracture hematoma, and lesser soft tissue damage can lead to earlier union in patients operated on with IMSCNs. Implant selection was also comparable to the studies in which the majority showed that DFCLP was the preferred implant for intercondylar distal femur fractures (intra-articular and highly comminuted fractures) in which open reduction, stable fixation, and anatomic reduction are achieved with ORIF [7,8,9,19,22]. IMSCN is the preferred implant for supracondylar (extra-articular) distal femur fractures. Our study also confirmed the same. Our results were also comparable to the studies on knee range of movement (ROM) (Schatzker and Lambert criteria), which was comparably equal for both groups after a follow-up of six months, with DFCLP patients having slightly better knee ROM than IMSCN patients [15,28]. In the IMSC nailing approach, splitting the patellar tendon through the knee joint is a disadvantage that affects the post-operative knee ROM of patients. Anterior knee pain was present in our series and was comparable to other studies [10-14]. 

Average blood loss was higher in the patients operated on with DFLCP, as shown in our study; 25% (5) of patients needed an intra-operative blood transfusion, of which four patients were operated on with DFLCP, while only one patient was operated on with an IMSCN. Open reduction, more soft tissue handling, and prolonged surgical time are the reasons for intraoperative blood transfusions in the DFLCP group. The results are comparable to the studies by Said et al. and Neradi et al. [28,30]. We used Schatzker and Lambert's criteria for the final analysis. The DFCLP group had excellent results in 46%, good and fair in 23% each, and only one patient had a poor outcome. For the IMSCN group, we found excellent and good results in 42.9% of each. One patient had fair results, and no patient had poor results, but the difference was found to be statistically insignificant. These results were comparable to the studies by Said et al. and Neradi et al. [28,30] and did not show the superiority of one implant over another.

We had two patients with open grade 3B distal femur intercondylar fractures, which were primarily operated on with an external fixator, and then definite fixation was done with DFCLP. Both showed good results at the final follow-up, showing that soft tissue compromise due to open fracture can affect the functional outcome like knee ROM and wound healing compared to closed fractures. Khan et al. showed comparable results in their study [29]. Neradi [30] conducted a meta-analysis that included pain, surgical duration, blood loss, implant failure, infection, knee range of motion, malunion, non-union, and union time. Blood loss (p <0.01) and surgery duration (p <0.01) favored the plating group, and the difference is significant. When other parameters such as knee ROM, implant failure, union time, and non-union were compared, our analysis favored the nailing group, but the difference was not significant. It shows there is no superiority of one implant over another. The main limitation of our study was the small sample size and a limited period of follow-up. A larger sample size with long-term follow-up could have provided a better comparison.

## Conclusions

We conclude that DFCLP is a better implant for inter-condylar (intra-articular) and highly comminuted distal femur fractures. It gives anatomic and stable fixation. The average intraoperative blood loss is higher with DFLCP. The supracondylar nail is the choice of implant for extra-articular distal femur fractures. The average union time and blood loss are less with IMSCN. Closed reduction, preservation of fracture hematoma, and less soft tissue compromise result in early fracture union in this group. Lack of rotational control, posterior fracture site angulation, damage to articular cartilage of the distal femur, and anterior knee pain are the negative points of IMSCN. Soft tissue compromise in open grade 3B fractures affects the functional outcomes. Following all surgical steps systematically avoids complications in both groups. Functional and clinical results do not change at the final follow-up with the use of either implant.
